# A Review: Multi-Omics Approach to Studying the Association between Ionizing Radiation Effects on Biological Aging

**DOI:** 10.3390/biology13020098

**Published:** 2024-02-04

**Authors:** Nathan A. Ruprecht, Sonalika Singhal, Kalli Schaefer, Om Panda, Donald Sens, Sandeep K. Singhal

**Affiliations:** 1 Department of Biomedical Engineering, University of North Dakota, Grand Forks, ND 58202, USA; nathan.ruprecht@und.edu (N.A.R.);; 2 Department of Pathology, University of North Dakota, Grand Forks, ND 58202, USA; 3 Department of Public Health, University of California Irvine, Irvine, CA 92697, USA

**Keywords:** ionizing radiation, biological age, multi-omics, bioinformatics

## Abstract

**Simple Summary:**

The effects of radiation exposure seem closely related to effects of old age—so much so that the idea of a radiation–age association came about in the 1960s. While not a new idea, modern technology is allowing us to revisit these ideas and explore them with a fresh perspective. Separately, there are gaps in the community’s understanding of the effects of radiation and aging, such as with respect to low-level, long-term effects of radiation and estimating someone’s biological age. To study their association, a number of tools exist that need to be efficiently integrated to study this complex and interdisciplinary field. This article includes an extensive literature review on the theory of these two topics, providing a detailed foundation for a current understanding. We then present a resource-agnostic approach for researchers in these areas, focusing on studying the association between the two. Primary points of interest are focused on indirect damage of radiation exposure via oxidative stress within a cell, a comprehensive table of functional estimators for biological age, and using modern computational tools and biology to overlap fields of study to develop and exploit a rad–age association.

**Abstract:**

Multi-omics studies have emerged as powerful tools for tailoring individualized responses to various conditions, capitalizing on genome sequencing technologies’ increasing affordability and efficiency. This paper delves into the potential of multi-omics in deepening our understanding of biological age, examining the techniques available in light of evolving technology and computational models. The primary objective is to review the relationship between ionizing radiation and biological age, exploring a wide array of functional, physiological, and psychological parameters. This comprehensive review draws upon an extensive range of sources, including peer-reviewed journal articles, government documents, and reputable websites. The literature review spans from fundamental insights into radiation effects to the latest developments in aging research. Ionizing radiation exerts its influence through direct mechanisms, notably single- and double-strand DNA breaks and cross links, along with other critical cellular events. The cumulative impact of DNA damage forms the foundation for the intricate process of natural aging, intersecting with numerous diseases and pivotal biomarkers. Furthermore, there is a resurgence of interest in ionizing radiation research from various organizations and countries, reinvigorating its importance as a key contributor to the study of biological age. Biological age serves as a vital reference point for the monitoring and mitigation of the effects of various stressors, including ionizing radiation. Ionizing radiation emerges as a potent candidate for modeling the separation of biological age from chronological age, offering a promising avenue for tailoring protocols across diverse fields, including the rigorous demands of space exploration.

## 1. Introduction

The tapestry of biological aging and the enigmatic interplay between ionizing radiation and the aging process have intrigued researchers across diverse scientific domains. As we delve deeper into the complex landscape of aging, the emergence of multi-omics approaches represents a promising avenue to dissect the molecular intricacies that underpin both natural aging and the influence of ionizing radiation. Through an exploration of the latest advancements and cutting-edge methodologies, this review navigates the exciting terrain of multi-omics, highlighting its potential to bridge the gap between radiation exposure and biological aging, ultimately reshaping our understanding of these intertwined phenomena.

The advent of multi-omics has revolutionized the study of ionizing radiation’s biological impact. Multi-omics integrates various omics fields, including genomics, transcriptomics, epigenomics, proteomics, and metabolomics, to provide a holistic view of cellular responses to radiation. Genomics identifies radiation-induced mutations and structural variations in DNA. Transcriptomics reveals changes in gene expression patterns, allowing for the identification of genes and pathways influenced by radiation exposure. Epigenomics investigates alterations in DNA methylation, histone modifications, and non-coding RNA expression, shedding light on epigenetic changes induced by radiation. Proteomics and metabolomics offer insights into the modulation of proteins and metabolic pathways, respectively, providing a deeper understanding of radiation’s effects on cellular function.

The quest to estimate biological age has a rich history, evolving from rudimentary measures to precise methodologies. Early aging estimators relied on basic indicators such as chronological age, body mass index, and blood pressure [1,2,3,4]. However, these initial approaches lacked the accuracy needed to capture the intricate nuances of aging. In recent decades, cutting-edge tools have emerged to assess biological age with unprecedented precision. Among these, DNA methylation-based clocks have revolutionized the field. Notable examples include the Horvath clock and the Hannum clock, which rely on DNA methylation patterns at specific CpG sites [5,6]. These clocks offer insights into an individual’s biological age, often differing from their chronological age, and serve as powerful predictors of aging-related outcomes. Myriad proteins, molecules, and enzymes play pivotal roles in the aging process. Among them, the tumor suppressor protein p53 is a guardian of genomic stability. It orchestrates responses to DNA damage, encompassing cell cycle arrest, DNA repair, and apoptosis, ultimately influencing the aging trajectory [7]. Another critical player is sirtuins, a family of NAD-dependent deacetylases that participate in the regulation of cellular functions, including DNA repair, metabolism, and stress response. These enzymes—particularly SIRT1—have been linked to longevity and are central to our understanding of the aging process [8]. Furthermore, molecules such as reactive oxygen species (ROS) and advanced glycation end products (AGEs) have garnered attention in the context of aging. ROS, generated during metabolic processes, can induce oxidative stress and contribute to cellular damage over time. AGEs, formed through the glycation of proteins and lipids, accumulate with age and can affect tissue function [9,10].

The integration of multi-omics approaches has ushered in a new era in the study of biological aging. Genomics, transcriptomics, epigenomics, proteomics, and metabolomics collectively provide a holistic view of aging-related processes. Genomics elucidates age-related genetic variations, including single-nucleotide polymorphisms (SNPs) associated with longevity and age-related diseases [11]. Transcriptomics, on the other hand, unveils changes in gene expression patterns during aging, shedding light on key pathways and regulatory networks involved in the aging process [12]. Epigenomics explores alterations in DNA methylation, histone modifications, and non-coding RNA expression, providing insights into epigenetic changes that accompany aging [13]. Proteomics and metabolomics offer a deeper understanding of the molecular alterations underlying aging. They uncover shifts in protein profiles and metabolic pathways, highlighting proteins and metabolites associated with aging-related phenotypes [14,15].

Ionizing radiation, a potent source of DNA damage, may intersect with the aging process. Recent research suggests that radiation-induced alterations in DNA methylation patterns and cellular senescence pathways may accelerate biological aging [16,17]. The bioinformatics aspect comes into play as researchers harness advanced computational tools to analyze multi-omics data generated from individuals exposed to radiation. Machine learning algorithms and network-based approaches that enable the identification of radiation-induced changes in gene expression, epigenetic markers, and protein profiles, shedding light on the molecular mechanisms underlying radiation-induced aging.

Research at the intersection of biological aging and radiation exposure holds promise. The use of advanced sequencing technologies such as single-cell RNA sequencing and epigenomic profiling provides high-resolution insights into the molecular events underlying aging and its potential intersection with radiation-induced processes. Moreover, personalized medicine approaches enable the tailoring of interventions based on an individual’s biological age and radiation exposure history. This precision medicine paradigm has the potential to mitigate health risks associated with radiation exposure and enhance resilience against aging-related diseases [18]. The discovery of a significant association between radiation exposure and biological aging holds profound implications across various fields and applications. Below, we present some key use cases and fields where such findings can have a transformative impact.

1. Radiobiology and radiation safety: Understanding how radiation exposure accelerates biological aging could lead to improved safety guidelines and radiation protection strategies for nuclear industry workers, astronauts, and healthcare professionals who frequently encounter ionizing radiation.
2. Space exploration: With human missions to Mars and beyond on the horizon, identifying the link between radiation exposure and aging is crucial for safeguarding the health of astronauts during extended space travel. Strategies to mitigate radiation-induced aging effects can be developed.
3. Health care and clinical practice: Radiation therapy is a cornerstone of cancer treatment. If radiation exposure is found to exacerbate biological aging, personalized radiation treatment plans can be devised to minimize long-term aging-related risks in cancer survivors.
4. Aging research and longevity: Discovering the connection between radiation and aging could unlock novel pathways for anti-aging interventions. This could lead to the development of pharmaceuticals, lifestyle interventions, and therapies to mitigate the effects of both natural aging and radiation-induced aging.
5. Environmental health: Studying the effects of radiation exposure on biological aging has implications for assessing and mitigating the health risks associated with nuclear accidents, such as Chornobyl and Fukushima, and long-term exposure in areas with elevated natural background radiation.
6. Public policy and regulation: Findings related to radiation exposure and aging may inform policy decisions regarding occupational exposure limits, environmental radiation standards, and radiation safety guidelines.

In essence, uncovering an association between radiation exposure and biological aging would not only deepen our understanding of fundamental biological processes but also have far-reaching implications for health care, space exploration, radiation safety, and longevity research, ultimately benefiting both individuals and society as a whole. The keys to understanding ionizing radiation effects are found in molecular biology. Much like any other aspect, conditions often overlap in how they interact or create disturbances within an organism. While ionizing radiation is more extreme in damage and mortality, it shares common themes with oxidative stressors in terms of what is happening to the cell, tissue, and organism, as well as the possible prognosis of developed diseases. Although not concrete, there is continual research and theory on the overlap in effects of radiation with those of natural aging of an organism. With a recent explosion of interest in biological age and expanding the lifespans of organisms, finding a modeling technique can aid in aging research. Especially with today’s technology, emerging fields like bioinformatics and computational biology are aiding in these studies with an eloquent combination of statistical techniques and model organism experiments [19,20]. Although there is some benefit to current occupations, mitigating procedures are in place that nearly negate the personnel’s exposure; the primary benefit of continuing to study the effects of oxidative stress on cells is for the next push in space exploration. The purpose of this paper is to review the biology side of ionizing radiation to create a solid foundation for future developments of industrial, academic, and government interest. The remainder of this paper is divided into two main sections, providing a bottom-up look at physiological changes feeding into research needs as applied to today’s gaps in understanding and tools.

## 2. Physiological Effects of Radiation

**Figure 1:**
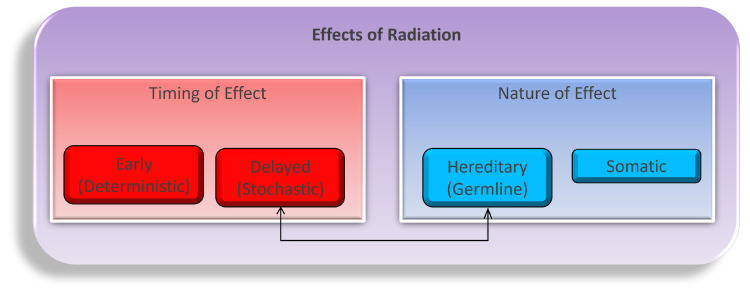
The flow of variables to consider when studying the biological effects of ionizing radiation on an organism. Both time and the nature of the radiation and the organism are shown as the first blocks in the breakdown. Underneath time are the short-term (early) and long-term (delayed) responses to radiation as the effects may set in over time. The germline and somatic conditions of the organism before and after radiation may also differ. Both sides of the flow chart lead to either cancer or other major health concerns within somatic cells [24].

Navigating the intricate landscape of ionizing radiation’s physiological effects is a multifaceted and dynamic endeavor, encompassing a complex interplay of temporal dimensions (early or delayed) and the nature of impacts (hereditary or somatic), as illustrated in Figure 1. These effects unfold through both direct and indirect mechanisms as radiation energy traverses the intricate terrain of tissues and cells. While the task of modeling the myriad consequences of these diverse combinations, encompassing variations in time frames, dosages, tissue types, organism characteristics, and the inherent nature of these effects, is undeniably intricate; it stands as an imperative pursuit in our quest to fathom the entirety of radiation’s influence [21,22,23]. Primarily, our concerns regarding the repercussions of radiation exposure gravitate towards somatic tissues, distinguished by their higher proliferation rates, with a pronounced emphasis on the intricate biology of blood and blood-forming organs, which constitute primary focal points of study. While the concept of directly induced damage, firmly situated within the realm of early timing and somatic nature, offers a more tangible and straightforward conceptualization, we must vigilantly acknowledge the nuanced complexities arising from indirect damage, as they intricately weave additional layers into the multifaceted tapestry of radiation’s impact on biological systems.

### 2.1. Generation of Reactive Species

One of the fundamental biological issues arising from ionizing radiation exposure is the generation of reactive oxygen species (ROS) and other reactive molecules. Ionizing radiation imparts energy to biological molecules, causing the ionization of atoms within these molecules. This process can lead to the formation of free radicals, which are highly reactive species due to their unpaired electrons. Notably, the hydroxyl radical (${\;}^{•}OH$) is a potent and highly reactive free radical generated during radiation exposure [25]. Its formation occurs through a complex series of chemical reactions, involving the radiolysis of water ($H_{2}O$) and the subsequent interaction of electrons and water molecules, as follows:

(1) $$\begin{array}{c} \mathrm{Radiolysis}\;\mathrm{of}\;\mathrm{water}:\;H_{2}O\overset{\mathrm{Radiation}}{\to }H^{•}+OH^{•}\\ \mathrm{Hydroxyl}\;\mathrm{radical}\;\mathrm{formation}:\;H^{•}+OH^{•}\overset{k}{\to }H_{2}O_{2} \end{array}$$

The resulting hydrogen peroxide ($H_{2}O_{2}$) is another reactive molecule with implications for cellular damage. Both ${\;}^{•}OH$ and $H_{2}O_{2}$ can initiate a chain reaction of oxidative stress, damaging cellular components, including DNA. The biological consequences of this damage or radiation are extensive and can manifest in various forms, including single-strand breaks (SSBs), double-strand breaks (DSBs), and base damage. Radiation-induced DNA damage can disrupt the genetic code, impair DNA replication, and lead to mutations, potentially contributing to cancer and other diseases. Single-strand breaks (SSBs) involve the rupture of one of the two complementary DNA strands. These breaks can result from the direct ionization of DNA or secondary reactions triggered by ROS. For example, the ${\;}^{•}OH$ radical causes the cleavage of the DNA strand as follows:

$$\mathrm{Deoxyribose}-H+OH^{•}\to {\mathrm{Deoxyribose}}^{•}+H_{2}O$$

The resulting sugar radical can then react with molecular oxygen ($O_{2}$), generating a ${\;}^{•}OH$ radical and causing the cleavage of the DNA strand as follows:

$${\mathrm{Deoxyribose}}^{•}+O_{2}\to OH^{•}+O_{2}^{-}\to OH^{-}+O_{2}^{•}\to H_{2}O$$

DSBs are particularly severe forms of DNA damage, as they involve the simultaneous breakage of both DNA strands. While direct ionization of DNA can lead to DSBs, they can also arise through the interaction of SSBs on opposing strands [26]. This interaction results in the formation of a DSB intermediate, which can be challenging to repair correctly. DSBs are particularly problematic because they can lead to chromosomal rearrangements and genomic instability, contributing to cancer development. At low doses of radiation (breakage is proportional to the equivalent dosage received), DSBs are estimated to occur in roughly 3% of cells, with breakage being proportional to the absorbed dosage. This is not enough to cause cell death; instead, 20–80 DSBs are needed to induce death.

Ionizing radiation can also induce damage to DNA bases, leading to base modifications or chemical alterations. For instance, the generation of ROS can result in the oxidation of DNA bases, including the conversion of guanine (*G*) to 8-oxo-guanine. This modification can disrupt base pairing during DNA replication and repair. If replication occurs before DNA repair or even in the event of inefficient repair, replication may not finish, causing permanent lesions, lethal damage to the cell, and apoptosis [27,28,29]. If this lesion is in the form of a base alteration or deletion, it could lead to long-term health issues associated with radiation exposure (most notably, cancer).

The physiological effects of radiation-induced DNA damage extend beyond the molecular level, profoundly impacting cellular function and homeostasis. DNA repair mechanisms such as base excision repair (BER) and homologous recombination (HR) are activated to rectify damaged DNA. However, when DNA damage overwhelms the repair capacity, cells may undergo apoptosis or cellular senescence, two distinct pathways that help maintain genomic integrity. Apoptosis, or programmed cell death, eliminates cells with severe DNA damage to prevent their propagation. Cellular senescence, on the other hand, induces a state of irreversible growth arrest, preventing damaged cells from dividing. However, the consequences of radiation-induced DNA damage are not limited to individual cells. These effects can ripple through tissues and organisms, leading to acute radiation syndrome (ARS) in severe cases or contributing to the long-term risk of cancer development. ARS encompasses a range of symptoms, including nausea, vomiting, diarrhea, and lethargy, and can be life-threatening.

### 2.2. Mitochondria Dysfunction

Within the intricate tapestry of radiation’s impact on biological systems, a central nexus emerges: the mitochondria. While the nucleus and nuclear DNA are undoubtedly primary targets of radiation damage, it is within the mitochondria that the initial seeds of disruption are often sown [30,31]. These essential organelles, often hailed as cellular powerhouses, wield significant influence over the deleterious effects of radiation. This influence stems from the unique vulnerability of mitochondrial DNA (mtDNA) to ionizing radiation, coupled with its comparatively limited repair mechanisms when contrasted with nuclear DNA [32]. Recent research employing high-resolution sequencing technologies has offered a nuanced view of mtDNA damage patterns induced by radiation. These studies have highlighted distinct lesions and structural alterations in mtDNA, shedding light on the mechanisms by which radiation impairs mitochondrial function.

Despite their distinctiveness, there exist intriguing similarities between nuclear and mitochondrial DNA repair pathways, and the overlap with aging processes adds a layer of complexity and interconnectedness to this paradigm [33,34,35]. Consequently, an examination of the radiation-induced damage to mitochondria invariably intersects with the broader context of biological aging, enriching our understanding of both phenomena.

The repercussions of mitochondrial damage are far-reaching, extending well beyond the confines of the organelle itself. Once mitochondrial function falters, the cell loses its primary source of energy, setting the stage for the development of metabolic diseases [36,37,38]. The mitochondrion’s substantial role in cellular respiration, where it consumes approximately 90% of the body’s oxygen, makes it a major contributor to the generation of reactive oxygen species (ROS) [39]. ROS, including superoxide ($O_{2-}^{•}$) and hydrogen peroxide ($H_{2}O_{2}$), are key players in oxidative stress, further exacerbating cellular damage and contributing to the aging process.

A pivotal component of understanding radiation-induced mitochondrial dysfunction lies in comprehending the electron transport chain (ETC). The ETC, comprising complexes I to IV within the inner mitochondrial membrane, along with coenzyme Q (CoQ) and cytochrome C (CytC), represents the heart of mitochondrial energy production [40]. Its primary purpose is to facilitate the breakdown of NADH and FADH2 into NAD and FAD, respectively, while producing free electrons and protons to establish a proton gradient essential for ATP synthesis. In the final act, Complex IV combines electrons, protons, and free oxygen to create harmless water molecules. However, radiation exposure introduces a disruptive element, as it can energize these electrons or water molecules, leading to the formation of the oxidative stressors previously mentioned. Recent research has offered a detailed view of the ETC’s intricate molecular machinery and the specific sites within it where radiation-induced disruptions occur. High-resolution structural biology techniques, including cryo-electron microscopy and X-ray crystallography, have unveiled the spatial organization of ETC complexes and their interactions with mitochondrial components [41].

Given the multifaceted challenges posed by radiolysis of water and ROS generation in mitochondria, the human body employs a multi-layered defense and repair strategy. Reactive measures aimed at mitigating the impact of free radicals often involve the use of antioxidants, whether from natural sources or as therapeutic interventions. While antioxidants offer a degree of protection, they fall short of providing a comprehensive and sustainable solution for long-term success [42]. The study of natural antioxidants as potential mitigators of ionizing radiation toxicity has been a topic of investigation for decades, with a consensus emerging that a combination of antioxidants can provide some level of protection, particularly in radiosensitive organs. However, a complete solution remains elusive [43,44,45,46]. Beyond traditional antioxidant strategies, recent investigations have explored emerging paradigms such as mitochondrial-targeted antioxidants and small molecules that modulate mitochondrial function.

### 2.3. Cellular Response

Studies have shed light on the molecular events underlying the cellular response to radiation-induced DNA damage, offering insights that have significant implications for both cancer treatment and radiation protection strategies. One key area of recent research has focused on the dynamic nature of DNA repair processes. Traditional models assumed a uniform and sequential repair of DNA lesions, but advanced genomic and proteomic techniques have uncovered a surprising level of complexity and heterogeneity [47]. Single-cell genomics has allowed scientists to dissect the DNA repair kinetics at the single-cell level, revealing that individual cells within a population can exhibit vastly different repair trajectories in response to radiation exposure [48]. This heterogeneity highlights the need for personalized approaches in cancer therapy, as understanding the repair kinetics of each patient’s tumor cells becomes increasingly crucial. Moreover, the crosstalk between different DNA repair pathways has garnered significant attention. Recent studies have elucidated the existence of a DNA repair “decision-making” network within cells [49]. This network prioritizes specific repair pathways based on the type and extent of DNA damage, ensuring that the most appropriate and efficient repair mechanisms are employed. The coordination of pathways such as non-homologous end joining (NHEJ), homologous recombination (HR), and base excision repair (BER) has unveiled a level of sophistication in the cellular response to DNA damage that was previously underestimated.

Advancements in structural biology have also provided a glimpse into the three-dimensional organization of DNA repair complexes. Cryo-electron microscopy and X-ray crystallography have enabled researchers to visualize the architecture of repair proteins and their interactions with DNA. This structural insight offers a platform for the design of targeted therapies that can modulate specific repair pathways, potentially enhancing the efficacy of radiation therapy or mitigating radiation-induced damage in healthy tissues [50] Furthermore, recent studies have delved into the interplay between DNA repair and chromatin structure. Radiation-induced DNA damage occurs within the context of chromatin, which can either facilitate or hinder the access of repair machinery to damaged sites [51]. Epigenetic modifications and chromatin remodeling have emerged as critical factors influencing DNA repair efficiency [52]. Understanding these epigenetic signatures and their impact on DNA repair provides a promising avenue for the development of interventions that can sensitize cancer cells to radiation therapy or protect normal tissues from radiation damage.

The three major mechanisms of DNA repair are a direct reversal of damage, base or nucleotide excision repair, and post-replication repair. These mechanisms have evolved and become very efficient for what humans are naturally exposed to [53,54]. A common research example is that of pyrimidine dimers, which are induced by ultraviolet light forming covalent linkages between adjacent thymine [55]. The solution is a photoreactivation where an enzyme cleaves the damaged area, allowing DNA polymerase to use the complement strand for new DNA molecules. The body has layers of redundancy and repair mechanisms for a plethora of damage types like this because of what already exists in a living environment. With regard to the discussed types of ionizing radiation, these mechanisms are not necessarily enough, and greater damage can occur.

### 2.4. Localized Effects

Zooming in on the diverse array of cell and tissue types impacted by ionizing radiation, a distinct pattern emerges, particularly at lower radiation doses. Cells that exhibit greater radiosensitivity typically share key characteristics, including undifferentiated status, a heightened proliferation rate, and elevated metabolic activity [56]. This observation aligns seamlessly with our earlier discussions, as cells engaged in active replication are inherently more vulnerable to the detrimental effects of ionizing radiation. Additionally, cells abundant in mitochondria face an elevated risk due to the production of reactive oxygen species (ROS) within these organelles. Notable examples of radiosensitive cell types encompass blood cells, skin cells, gastrointestinal cells, and sperm cells, while nerve cells and muscle fibers display higher resistance. The spectrum of illnesses stemming from ionizing radiation exposure mirrors the diverse range of affected cells and organs, both directly and indirectly.

While immediate concerns revolve around acute radiation syndrome, predominantly induced by high doses of radiation administered over a short duration, lower radiation doses give rise to intermediate and long-term health concerns, most prominently cancer. Singularly focusing on mitigating a single radiation-induced illness is an incomplete approach, given the intricate interconnectedness of the human body’s responses to radiation exposure. Moreover, there is a substantial overlap between diseases attributed to ionizing radiation exposure and age-related conditions. In both scenarios, patients face an elevated risk of developing cardiovascular diseases, osteoporosis, and neurological disorders such as Alzheimer’s disease [57,58,59,60]. This convergence of radiation-related and age-related research fields provides fertile ground for collaborative efforts. Age can be perceived as the cumulative result of DNA damage accrued over time, while cancer predominantly results from DNA mutations. The cumulative effect of these conditions suggests heightened radiosensitivity, potentially compounded by genetic predispositions.

As researchers delve into the complex arena of transgenerational effects stemming from irradiated parents, a consensus emerges, albeit within a complex landscape. Phenotypic variations attributed to genetic composition often confound interpretations [61,62,63]. However, a notable agreement centers on the distinction between irradiated males and females concerning the transmission of germline mutations and potential somatic mutation rate alterations through sperm [64,65]. While substantial progress has been made in comprehending cellular responses and sensitivity to radiation since the 1980s, a substantial clinical gap remains in accurately predicting the likelihood of severe damage based on individualized histories [66,67,68,69,70].

Promisingly, emerging techniques empowered by contemporary computational capabilities hold great potential for advancing our understanding of radiation-induced effects, encompassing neurological impacts. New methodologies include next-generation sequencing, multi-omics integration, and the application of machine learning and artificial intelligence to decipher intricate patterns within this multifaceted landscape. These innovative approaches not only promise to refine our comprehension of radiation-induced cellular and molecular responses but also hold the potential to illuminate the neurological effects of radiation exposure, ushering in a new era of precision in radiation science and health care.

### 2.5. Effecting the Microbiome

Ionizing radiation, while primarily recognized for its impact on human cells, extends its reach to the intricate world of the human microbiome, an ecosystem teeming with microbial life that plays a pivotal role in maintaining human health. With an estimated 10 times as many microbial cells as human cells in the body, the human microbiome encompasses the diverse array of microbes residing on or within various tissues and fluids. It is a dynamic landscape, where even short distances can yield significant variances, as evidenced by experiments collecting samples from different facial areas, like the nose and lips. Disturbances in the delicate balance of this microcosm can contribute to a spectrum of diseases, including depression, autism spectrum disorders, obesity, diabetes, cancer, infections, and many others [71,72,73,74].

The radiosensitivity of these microbes, coupled with their predominant residence within the gastrointestinal tract, adds complexity to the puzzle of understanding radiosensitivity in humans. The potential for dysbiosis, wherein certain microbes may survive radiation exposure within the GI tract, raises intriguing questions about the consequences of such microbial resilience. Research has even unveiled instances where certain microbes indirectly protect their host from the harmful effects of radiation, as their survival contributes to the well-being of multiple organ systems [75,76].

Microbial DNA, much like its human counterpart, is susceptible to the effects of ionizing radiation, both through direct and indirect mechanisms. While delving into the specifics of radiation effects on microorganisms is a vast undertaking beyond the scope of this paper, it is essential to recognize the significance and sheer magnitude of the field. Understanding the survivability and post-radiation effects on this crucial component of human homeostasis is an ongoing area of research [77,78,79].

The microbiome represents one of the integral pieces of the puzzle that must be considered in the broader context of radiation’s impact on the entire organism. Its intricate interplay with human health and its potential to influence and be influenced by radiation effects necessitate comprehensive research efforts. Recent scientific advancements in microbiome research have unveiled novel insights into how ionizing radiation perturbs this delicate ecosystem and how changes in microbial composition can, in turn, affect host physiology. These discoveries hold the promise of improving our ability to predict and mitigate radiation-induced health effects by considering the microbiome as an integral player in the complex symphony of radiation response.

## 3. Biological Aging Theory

A cornerstone in the exploration of the intricate relationship between ionizing radiation and biological aging is the free radical theory of aging proposed by Denham Harman in 1955. Harman’s theory posits that the aging process is characterized by the cumulative oxidative damage inflicted upon cells by free radicals [80]. However, the landscape of aging theories is far from static, and contemporary perspectives have expanded beyond the notion that oxidative damage alone drives aging. Instead, it is increasingly recognized that aging is a multifaceted phenomenon shaped by a multitude of factors. While oxidative damage remains a fundamental contributor, it represents just one facet of the intricate web of processes culminating in cellular dysfunction and, ultimately, aging-related outcomes [81]. In this section, we delve into the evolving landscape of biological aging theory, encompassing not only oxidative stress but also factors such as inflammation, disease development, and multi-omic alterations that collectively contribute to the nuanced understanding of the aging process.

### 3.1. Hallmarks and Indicators

In 2013, López-Otín and colleagues introduced a foundational framework known as the “nine hallmarks of aging” that provided a comprehensive overview of the key processes and pathways contributing to the aging phenomenon [82]. These hallmarks serve as a crucial starting point for understanding the molecular intricacies of aging, and they encompass a broad spectrum of biological phenomena that impact the aging process. In this exploration, we not only delve into these hallmarks but also delve deeper into the genetic, epigenetic, and proteomic biomarkers that provide insights into the aging process, offering a glimpse into recent scientific advancements in this ever-evolving field.

**Genomic Instability**: At the core of aging lies genomic instability, characterized by accumulating DNA damage and mutations over time. Recent advances in genomics have unveiled a plethora of genes intricately involved in DNA repair and maintenance. Notable genes such as *P16, P21, FOXO1, P53, SIRT1, SIRT6, TNFa, IL6, TFAM, GATA6, ALOX15B, MAOA, TSC1, mTOR, RIPK1, RIPK3*, and *MLKL* have emerged as central players in safeguarding genomic integrity. Moreover, modern technologies like next-generation sequencing have empowered researchers to explore the genomic landscape of aging with unprecedented precision, uncovering novel genetic factors and intricate pathways contributing to the aging process.

**Telomere Attrition**: Telomeres, the protective caps at the ends of chromosomes, erode with each cell division, serving as a cellular countdown clock. Recent breakthroughs in telomere biology have unveiled the roles of telomerase and shelterin complex proteins in preserving telomere length and stability. The identification of genetic and epigenetic factors influencing telomere maintenance has illuminated potential avenues for interventions to counteract telomere attrition and extend cellular lifespan.

**Epigenetic Alterations**: Epigenetics, the study of heritable changes in gene expression that do not involve alterations to the underlying DNA sequence, plays a pivotal role in aging. Recent research has spotlighted DNA methylation as a prominent epigenetic marker of aging. Advances in epigenome-wide association studies (EWASs) have identified specific methylation patterns associated with aging, paving the way for the development of epigenetic clocks that accurately estimate an individual’s biological age. MicroRNA research, while promising, remains an area ripe for further exploration to harness its full potential in unraveling the epigenetic intricacies of aging.

**Loss of Proteostasis**: Maintenance of proteostasis, i.e., the balance between protein synthesis, folding, and degradation, is essential for cellular health. Recent studies have highlighted the roles of chaperones, autophagy, and the ubiquitin–proteasome system in maintaining proteostasis. Advancements in proteomic techniques have enabled the identification of specific protein aggregates and misfolded proteins associated with age-related diseases, shedding light on potential therapeutic targets to mitigate proteostatic decline.

**Deregulated Nutrient Sensing**: The deregulation of nutrient-sensing pathways, including insulin and *mTOR* signaling, contributes to aging-related metabolic dysfunction. Recent research has elucidated the roles of genes like *FOXO*, *AMPK*, and *SIRT1* in nutrient sensing and longevity. Additionally, proteomic studies have unveiled key proteins involved in nutrient-sensing pathways, providing insights into their intricate regulation and potential interventions to modulate nutrient sensing in aging.

**Mitochondrial Dysfunction**: Mitochondria, the cellular powerhouses, play a pivotal role in aging through their influence on energy production and the generation of reactive oxygen species (ROS). Recent advancements in mitochondrial research have unveiled the intricate mechanisms underlying mitochondrial dysfunction, including mitochondrial DNA mutations and impaired electron transport chain activity. Novel mitochondrially targeted therapies and antioxidants hold promise in mitigating the age-associated decline in mitochondrial function.

**Cellular Senescence**: Cellular senescence, characterized by irreversible cell cycle arrest, contributes to aging-related tissue dysfunction. Recent discoveries have illuminated the roles of the senescence-associated secretory phenotype (SASP) and specific senescence-inducing genes in driving senescence-related inflammation and tissue deterioration. Proteomic investigations have identified key SASP components, offering potential targets to modulate the detrimental effects of cellular senescence.

**Stem Cell Exhaustion**: Stem cell exhaustion, a hallmark of aging, limits tissue regeneration and repair. Recent advances in stem cell biology have unraveled the genetic and epigenetic mechanisms governing stem cell function. Techniques such as single-cell RNA sequencing have provided insights into the heterogeneity of stem cell populations and their responses to aging-related cues. Strategies to rejuvenate or replace exhausted stem cells hold promise for rejuvenation therapies.

**Altered Intercellular Communication**: Age-related changes in intercellular communication through factors like cytokines and growth factors contribute to aging-related pathologies. Recent studies have unveiled the roles of specific genes and pathways in mediating intercellular communication in aging. Proteomic approaches have identified key signaling molecules and their dysregulation in aging-related diseases, offering potential avenues for targeted interventions.

In addition to these hallmarks, metabolomics has emerged as a burgeoning field poised to provide comprehensive insights into the metabolic changes associated with aging and age-related diseases. Metabolomic profiling, combined with genetic, epigenetic, and proteomic data, promises a holistic understanding of the aging process, enabling the identification of biomarkers and therapeutic targets to enhance healthy aging and extend lifespan.

### 3.2. Overlapping Health Concerns

The intricate interplay between aging, radiation exposure, and associated health concerns transcends age groups, encompassing individuals across the entire lifespan. Recent advancements in medical research have unveiled trends and disparities in the impact of aging and radiation on health, shedding light on varying susceptibilities and implications in both young and older populations. Here, we delve into the multifaceted relationship between aging, radiation exposure, and age-related diseases, emphasizing trends observed in different age groups and incorporating relevant citations.

Inflammation and Aging Across Age Groups: Chronic inflammation, a hallmark of aging, exhibits varying trends across different age groups. In younger individuals, acute inflammation is often a protective response to infections and injuries. However, in older adults, chronic low-grade inflammation, known as “inflammaging”, becomes increasingly prevalent. Recent research, as cited by Podolskiy et al. and Manoharan et al., highlights the role of inflammation in age-related diseases across age groups. For instance, neurodegenerative conditions like Alzheimer’s, Huntington’s, and Parkinson’s diseases exhibit distinct trends in younger versus older populations, with genetic and epigenetic factors contributing to these disparities [83,84,85].

Cardiovascular Health Across Age Groups: Cardiovascular diseases, a common consequence of aging, can manifest differently in various age groups. Recent advancements in cardiology, as mentioned by Curtis et al. and De et al., reveal age-specific trends in cardiovascular health. While older individuals are more susceptible to age-related cardiovascular changes, radiation exposure can exacerbate these trends, affecting both young and older populations. Understanding these nuances is crucial for effective cardiovascular disease management across age groups [86,87,88,89].

Autoimmune Diseases Across Age Groups: Autoimmune diseases, characterized by dysregulated immune responses, can affect individuals of all ages. Recent research cited by Goronzy et al. and Weck elucidates age-specific trends in autoimmune diseases. In pediatric populations, autoimmune conditions may have distinct genetic and environmental triggers, while older adults may experience age-related changes in immune function that contribute to autoimmune pathogenesis. Understanding these age-related disparities is crucial for tailored diagnosis and management across age groups [43,90,91].

Cancer Across Age Groups: Cancer, often considered a hallmark of aging, exhibits variations in incidence and pathogenesis across different age groups. Recent genomic studies, as noted by Podolskiy et al., have unveiled age-specific genetic alterations underlying various cancer types. While older individuals are more prone to age-related genomic changes, radiation exposure can influence cancer development in both young and older populations. Research into these age-related trends is essential for personalized cancer care across age groups [83,92].

Other Health Concerns Across Age Groups: Age-related health concerns extend beyond the aforementioned categories to encompass a wide range of conditions. Recent advancements in medical research have provided insights into age-specific trends in conditions such as cerebrovascular diseases, fibrosis, osteoporosis, chronic inflammation, hypertension, and cataracts. These trends can vary significantly between young and older populations, emphasizing the need for age-appropriate prevention and management strategies [87,89,93,94,95,96].

### 3.3. Estimating Biological Age

Associating oxidative stress with age presents a complex challenge, primarily because defining age as a baseline reference point is not straightforward. Chronological age, representing the number of years an individual has lived, is an uncontrollable factor that cannot be influenced to observe the effects of stressors. In contrast, biological age, which reflects an individual’s overall health and physiological state, cannot be directly measured or independently estimated. To navigate this complexity, researchers have developed gold standards for estimating biological age, aiming to bridge the gap between chronological and biological age.

The quest for accurate biological age estimation began in 1985 with Nakamura et al.’s pioneering work [97]. They conducted principal component analysis (PCA) of uncorrelated biomarkers and employed Hochschild’s method in 1989 to address the paradox of chronological age. Hochschild’s approach assumes that biological age depends on chronological age for regression analysis, laying the groundwork for subsequent developments in biological age estimation. Building upon Nakamura’s foundation, modern standards emerged, with the Klemera and Doubal (KD) method taking center stage in 2006 [1]. The KD method is widely regarded as the go-to approach for estimating biological age. It incorporates chronological age as one of the primary indicators and integrates it into the modeling process. Researchers such as Levine [2] and Cho [3] extended the KD method’s utility by employing it to predict mortality and supplementing it with factors like the Work Ability Index (WAI).

However, recent studies in the field of biological age estimation have expanded our understanding and refined our ability to separate chronological and biological age. One notable advancement is the development of epigenetic clocks, which utilize DNA methylation patterns to estimate an individual’s biological age [5]. These clocks, pioneered by Horvath in 2013, have demonstrated remarkable accuracy in predicting age, outperforming traditional methods in some cases. The epigenetic clock concept has since evolved, with various clock models tailored to different tissues and populations, enhancing our ability to estimate biological age with precision [6,98,99]. Additionally, recent research has explored the integration of multi-omic data, including genomics, epigenomics, transcriptomics, and proteomics, to develop comprehensive biological age estimators [100]. These integrative approaches consider a broader spectrum of molecular markers, providing a more holistic view of an individual’s biological age. Despite these advances, the pursuit of refined biological age estimation continues, fueled by the recognition that a more comprehensive understanding of aging and its associated oxidative stressors can yield valuable insights into health and longevity.

Indicators of biological age can be categorized into three major groups: functional, physiological, and psychological well-being (PWB). Each category provides valuable insights into the aging process and can help estimate an individual’s biological age more accurately. Table 1 presents a comprehensive list of potential biomarkers within these categories, highlighting their significance in assessing the extent of cellular damage and repair, thereby contributing to a more precise estimation of biological age. This table is a unique contribution to aging research, since it is the first holistic overview of markers including the psychological aspect. Here, we highlight the required measures to tackle complete and accurate estimation of biological age, allowing individualized studies to dive deeper into how to measure each entry via targeted biomarkers. An argument is that no study to date has completed its objective in setting a reference for BA estimation, since these categories have not been sufficiently explored.

- Functional biomarkers encompass both cognitive and physical aspects of an individual’s health. These biomarkers offer valuable insights into how well an individual’s mind and body are functioning. Within the cognitive realm, indicators such as memory, decision reaction time, and verbal fluency provide critical information about cognitive decline or preservation. On the physical front, biomarkers like grip strength, walking speed, and visual perception and measures like height, weight/BMI, and lung capacity offer insights into an individual’s physical vitality and resilience [101,102,103,104,105,106].
- Physiological biomarkers delve into the state of an individual’s organs, tissues, and cellular health. These biomarkers provide a deeper understanding of the body’s internal processes and can shed light on the effects of aging. Metrics such as brain size, blood composition, blood pressure, muscle mass, and bone density, among others, offer valuable data for assessing an individual’s physiological age [107,108].
- Psychological well-being biomarkers are a unique category that delves into an individual’s emotional and mental state. This category is further divided into hedonic and eudaimonic dimensions. Hedonic aspects focus on happiness, subjective well-being, and positive emotions, while eudaimonic dimensions include self-acceptance, environmental mastery, positive relationships, personal growth, purpose in life, and autonomy. These biomarkers offer insights into an individual’s psychological resilience and overall well-being, which can influence their biological age [112,113].

While these biomarkers provide a comprehensive foundation for estimating biological age, it is essential to recognize that the field of biological age estimation has evolved significantly in recent years. Mentioned earlier was the groundbreaking development of epigenetic clocks that have revolutionized the way we approach biological age estimation. These clocks utilize DNA methylation patterns to provide highly accurate estimations of an individual’s biological age. Pioneered by researchers like Horvath and Hannum, epigenetic clocks have showcased their remarkable precision and have become valuable tools in aging research [114,115]. The next significant developments coming in the near future involve the integration of multi-omic data, including genomics, epigenomics, transcriptomics, and proteomics, opening up new avenues for the development of comprehensive biological age estimators. These integrative approaches consider a wide range of molecular markers, offering a holistic view of an individual’s biological age and health status.

As the field continues to advance, the standard for biological age estimation continues to evolve, with new methodologies and interpretations constantly adding to our understanding of the aging process [116]. This progress is crucial for gaining insights into age-related diseases and their potential associations with radiation exposure. Developing optimized models and techniques for clear and meaningful results will expedite theoretical progress and enhance our ability to mitigate the effects of oxidative stressors on biological age.

## 4. Studying the Association

Studying the association between ionizing radiation exposure and biological aging is a complex and multifaceted task that demands cutting-edge scientific tools, meticulous analysis, and a deep understanding of the intricate molecular pathways that underlie both processes. This section navigates the landscape of research methodologies, delving into the current state of tools available for investigating this intricate connection. It explores the shared pathways and biomarkers that bridge the realms of radiation exposure and aging, shedding light on the underlying mechanisms that drive their association. Additionally, it investigates the burgeoning field of multi-omics and modeling, which offers a comprehensive view of the biological landscape, allowing for a more nuanced understanding of the interplay between radiation and aging. We also delve into multivariable association research to disentangle the complex web of factors contributing to the observed effects. Finally, we glimpse the future, where ongoing developments promise to further illuminate this intriguing relationship, providing valuable insights into the potential health consequences of ionizing radiation exposure across the lifespan.

### 4.1. Current State of Tools

Researchers have harnessed a diverse array of tools and techniques that span the domains of biology, computational science, and data analysis. This multifaceted endeavor seeks to uncover the mechanisms by which radiation impacts the aging process, all while leveraging cutting-edge technologies to explore and quantify this intricate relationship. Here, we delve deeper into the current state of tools and techniques, emphasizing the role of artificial intelligence (AI) and machine learning (ML) in recent scientific advancements.

One of the most significant challenges faced in this endeavor is the management and analysis of vast biological datasets. These datasets are inherently complex, featuring intricate connections and dependencies that necessitate advanced computational approaches for meaningful interpretation. The conventional computer architecture, while powerful, has limitations when it comes to accurately representing this intricate web of biological connections. However, considerable progress is being made to adapt programming languages and data processing frameworks to accommodate the complexity of biological data. Tools such as Biopython and Bioinformatics provide researchers with the means to manipulate, format, and analyze large-scale biological datasets, paving the way for valuable insights [117,118].

A significant barrier for bioinformaticians is the accessibility of both data and the tools required to work with them effectively. The cost of infrastructure, including storage and processing power, is a critical consideration. Researchers are continually developing, evaluating, and iterating techniques for data mining, analysis, and interpretation. Bioinformatics, with its significant overlap and synonymity with computational biology, is evolving as a field of expertise, although it is not without its challenges and variability in conclusions [119,120]. However, recent developments in AI and ML have ushered in a new era for bioinformatics and computational biology. These technologies have proven invaluable in handling the complexity and scale of biological data. AI algorithms such as deep learning neural networks have demonstrated remarkable capabilities in pattern recognition and predictive modeling, enabling researchers to extract meaningful insights from vast datasets. For example, convolutional neural networks (CNNs) have been applied to image analysis in radiology, aiding in the detection and classification of radiation-induced tissue damage or aging-related changes in medical images [121]. This has far-reaching implications for studying the effects of radiation exposure on biological aging, as it allows for the precise quantification of structural and functional changes in tissues and organs.

Moreover, ML techniques have empowered researchers to predict biological age more accurately. The advent of the epigenetic clock, pioneered by Horvath [5], has revolutionized the field. This clock leverages DNA methylation patterns to estimate an individual’s biological age, providing a powerful biomarker for aging. Recent advancements in epigenetic clock research have led to the development of highly accurate models, such as the GrimAge clock [98], which incorporates additional DNA methylation markers associated with mortality and morbidity. These epigenetic clocks not only enhance our ability to estimate biological age but also enable us to investigate how radiation exposure may accelerate or decelerate the aging process at the molecular level. AI and ML techniques have been instrumental in identifying and characterizing specific pathways and biomarkers associated with radiation-induced aging. High-dimensional omics data, including genomics, transcriptomics, and proteomics, pose unique challenges in data analysis. However, ML algorithms such as random forests and support vector machines have proven effective in feature selection and classification tasks. Recent studies have utilized these techniques to identify key genes and proteins involved in radiation response and aging-related processes. For instance, the identification of genes associated with cellular senescence, oxidative stress response, and DNA repair mechanisms has shed light on the molecular underpinnings of radiation-induced aging [122,123].

Another notable advancement is the application of AI-driven radiomics in radiation biology. Radiomics involves the extraction of quantitative features from medical images, enabling the characterization of tissue properties and heterogeneity. By applying ML algorithms to radiomic data, researchers can uncover subtle changes in tissue composition and structure caused by radiation exposure. This approach has the potential to provide early indicators of aging-related effects, such as fibrosis or tissue degeneration, allowing for timely interventions [124]. AI and ML have found utility in predicting radiation therapy outcomes and optimizing treatment plans. These techniques consider a multitude of factors, including patient demographics, tumor characteristics, and treatment parameters, to tailor radiation therapy for improved efficacy and reduced side effects. Reinforcement learning algorithms, for example, can adapt treatment plans in real time based on patient response, ensuring the delivery of the most effective dose while minimizing damage to healthy tissues [125,126,127].

Along with algorithmic progress, clinical success is shifting how we approach rad–age applicability. A hormesis-based skincare cosmetic case study highlighted the favorable consequences of mild stress with respect to cellular repair mechanisms, aligning conceptually with ongoing research on the rad–age association [128]. Both investigations share a common objective in comprehending stress-induced molecular responses and their nuanced implications for the aging process. The efficacy of hormesis-based products, characterized by the induction of stress genes and proteins for anti-aging effects, provides valuable insights into potential interventions. Concurrently, as the study progresses to unveil the impact of ionizing radiation on biological aging, correlations with stress-induced beneficial outcomes may inform innovative strategies for mitigating radiation-induced aging. The spreading acceptance of hormesis-based approaches underscores a societal shift in recognition, establishing a pertinent context for the research to influence perspectives and guide healthcare strategies.

Conversely, another study looked at accelerated aging due to radiation exposure, emphasizing the community’s lack of understanding of the cost versus benefit of radiation usage. The study investigated the impact of prolonged radiation exposure in the catheterization laboratory (cath lab) upon early signs of subclinical atherosclerosis [129]. Examining carotid intima–media thickness (CIMT), leukocyte telomere length (LTL), and the XRCC3 Thr241Met polymorphism, the research found significantly increased CIMTs and reduced LTL in high-exposure workers. Left-sided CIMT correlated with occupational radiological risk scores and lifetime dose. The XRCC3 Met241 allele exhibited a substantial interaction with high exposure, influencing CIMTs. These findings, highlighting accelerated vascular aging and early atherosclerosis due to long-term radiation exposure, resonate indirectly with rad–age association research, providing insights into the connections between ionizing radiation, vascular aging, and potential accelerated aging processes.

Lastly, let us highlight that a rad–age association is not necessarily a new concept; the novelty lies in how modern approaches can better exploit it. A study conducted in 1976 investigated the lifespan of nine inbred and five hybrid mouse strains, exposing some to X-ray irradiation at varying doses [130]. The dataset, comprising 400 mice of each sex in the inbred group and 200 mice of each sex in the hybrid group, includes strain details, sample size, and mean survival times. While primarily to better understand the long-term effects of radiation, this early exploration into the effects on mouse lifespan underscores the longstanding nature of research in this domain, contributing insights that align with the historical context of rad–age association research.

### 4.2. Key Overlapping Biomarkers and Pathways

The overlapping biomarkers, pathways, and diseases associated with ionizing radiation exposure and natural aging represent a complex interplay of biological processes that provide valuable insights into the shared mechanisms and potential health risks. Understanding these commonalities is crucial for assessing the effects of radiation on aging and age-related diseases, as well as for the development of strategies to mitigate these risks. In this discussion, we delve into the intricate web of overlapping factors, highlighting recent studies and discoveries that shed light on this critical area of research.

One of the central pathways shared between ionizing radiation exposure and natural aging is the accumulation of DNA damage and its repair mechanisms. Both processes lead to an increase in DNA lesions, such as double-strand breaks and oxidative DNA damage. DNA damage response pathways, including the activation of DNA repair enzymes and cell cycle checkpoints, are triggered in response to radiation-induced damage, as well as age-related DNA lesions. Recent studies have identified specific biomarkers associated with DNA damage and repair that overlap between radiation exposure and aging. For instance, the tumor suppressor protein p53 plays a pivotal role in regulating the cellular response to DNA damage caused by radiation and age-related factors. Studies have shown that p53 activity is elevated in aging tissues and in response to radiation exposure, contributing to cell cycle arrest and apoptosis [131]. Telomere shortening is another hallmark of both natural aging and radiation-induced aging. Telomeres, protective caps at the ends of chromosomes, progressively shorten with each cell division. Radiation exposure can accelerate telomere attrition, leading to premature cellular senescence. Research has also highlighted the importance of telomere length as a biomarker of aging and radiation sensitivity [132].

Oxidative stress and chronic inflammation are critical pathways that link ionizing radiation exposure and aging-related diseases. Radiation exposure generates reactive oxygen species (ROS) as a result of radiolysis of water molecules. Similarly, during natural aging, there is an imbalance between ROS production and antioxidant defenses, leading to oxidative stress. Recent studies have provided compelling evidence of the role of oxidative stress in both radiation-induced and natural aging. Biomarkers such as 8-hydroxy-2’-deoxyguanosine (8-OHdG), a marker of oxidative DNA damage, have been identified in individuals exposed to radiation and in aged tissues. The upregulation of antioxidant enzymes such as superoxide dismutase (SOD) and glutathione peroxidase (GPx) serves as a common protective response against oxidative stress in both scenarios [133].

Inflammation is another shared pathway, as radiation exposure and aging can lead to chronic low-grade inflammation, often referred to as inflammaging. Recent research has elucidated the role of pro-inflammatory cytokines, such as interleukin-6 (IL-6) and tumor necrosis factor-alpha (TNF-α), in promoting inflammation during aging and in radiation-induced tissue damage. These cytokines contribute to age-related diseases such as cardiovascular disease, cancer, and neurodegenerative disorders, which are also influenced by radiation exposure [134].

Cellular senescence, characterized by irreversible cell cycle arrest, is a prominent feature of both natural aging and radiation-induced aging. Senescent cells accumulate in tissues over time, secreting a range of bioactive molecules collectively known as the senescence-associated secretory phenotype (SASP). SASP components include pro-inflammatory cytokines, growth factors, and matrix metalloproteinases, contributing to tissue dysfunction and age-related pathologies. Recent studies have highlighted the significance of cellular senescence and SASP in radiation-induced aging and natural aging. Biomarkers such as senescence-associated β-galactosidase (SA-β-gal) activity and p16INK4a expression serve as indicators of cellular senescence and have been detected in tissues exposed to radiation and in aging tissues. Targeting senescent cells and SASP components has emerged as a promising strategy for mitigating radiation-induced aging and age-related diseases [135].

The convergence of pathways and biomarkers in radiation exposure and natural aging contributes to the development of age-related diseases. These diseases, including cardiovascular disease, cancer, neurodegenerative disorders, and metabolic syndromes, are influenced by both processes. Recent studies have emphasized the role of radiation exposure as a risk factor for these diseases, particularly when combined with advancing age. Cardiovascular disease, characterized by atherosclerosis and cardiac dysfunction, is a prime example of an age-related disease exacerbated by radiation exposure. Recent research has elucidated the role of radiation-induced oxidative stress and inflammation in promoting vascular damage and atherogenesis. Biomarkers such as C-reactive protein (CRP) and endothelial dysfunction markers have been identified as shared indicators of cardiovascular risk in radiation-exposed populations and aging individuals [136,137]. Furthermore, the overlap between radiation exposure and aging in the context of cancer is a subject of intense investigation. Recent studies have revealed the influence of radiation-induced DNA damage, telomere dysfunction, and cellular senescence on cancer risk. Biomarkers such as oncogenes, tumor suppressor genes, and specific genetic mutations have been identified as common factors contributing to both radiation-induced carcinogenesis and age-related cancer development. Understanding the shared pathways and biomarkers has prompted efforts to refine cancer screening and prevention strategies for both radiation-exposed populations and the elderly.

This section is not all-inclusive. The sheer amount of studies that exist exploring each gene, each enzyme, and each disease, while creating complex biological connections and speculating about confidence in results, would all be out of scope and unfair to the readers here. Instead, the point to be made is recognizing and understanding key processes that should be emphasized when conducting rad–age studies.

### 4.3. Multi-Omics and Modeling

Multi-omics and modeling approaches have played a pivotal role in confirming the association between ionizing radiation exposure and biological aging. These sophisticated techniques offer a comprehensive view of the molecular and cellular changes that occur as a result of radiation exposure, allowing researchers to identify key variables and mechanisms underlying the rad–age association. Recent advancements in multi-omics and modeling have significantly enhanced our understanding of this complex relationship.

One of the key areas where multi-omics has been instrumental is in elucidating the impact of radiation on the epigenome. Epigenetic modifications such as DNA methylation play a crucial role in regulating gene expression and have been linked to aging processes. Recent studies have employed techniques like DNA methylation arrays and next-generation sequencing to profile the epigenetic changes induced by radiation exposure. Horvath’s epigenetic clock was used to demonstrate accelerated aging in individuals exposed to radiation, particularly in the context of medical radiation therapy or nuclear accidents [138]. Advances in transcriptomics have allowed researchers to examine changes in gene expression patterns following radiation exposure. High-throughput RNA sequencing (RNA-seq) has enabled the identification of radiation-responsive genes and pathways. Studies have shown that radiation exposure can lead to dysregulation of genes involved in DNA repair, inflammation, and oxidative stress response. These findings highlight the molecular mechanisms through which radiation accelerates biological aging and increases the risk of age-related diseases [139]. Metabolomics has provided valuable insights into the metabolic changes associated with radiation exposure. Mass spectrometry and nuclear magnetic resonance spectroscopy have been used to profile metabolites in biological samples. These studies have revealed alterations in metabolic pathways related to energy production, oxidative stress, and cellular homeostasis. Notably, the accumulation of reactive oxygen species (ROS) and oxidative damage to lipids, proteins, and nucleic acids have been identified as central factors linking radiation exposure to accelerated aging [140].

The integration of multi-omics data has allowed for the construction of comprehensive models that capture the complex interactions between radiation-induced molecular changes and biological aging. Machine learning and artificial intelligence (AI) techniques have been employed to develop predictive models that assess an individual’s biological age based on multi-omics data. These models take into account variables such as DNA methylation patterns, gene expression profiles, and metabolic signatures to estimate the impact of radiation exposure on aging. Recent studies have demonstrated the utility of AI-based models in accurately predicting biological age and identifying radiation-induced aging signatures. For example, deep learning algorithms have been applied to multi-omics datasets to identify radiation-specific biomarkers associated with aging [141]. These biomarkers offer valuable insights into the biological processes affected by radiation and contribute to our understanding of the rad–age association. Building on top of this, systems biology approaches have been used to construct network models that depict the crosstalk between different omics layers and their influence on aging [142,143]. These models provide a holistic view of the molecular pathways affected by radiation exposure and how they converge with aging-related processes. Recent discoveries have highlighted the role of inflammation, DNA repair mechanisms, and cellular senescence as key nodes in these networks, shedding light on the interconnected nature of radiation-induced aging.

### 4.4. Challenges, Gaps, and Shortfalls

Studying the relationship between ionizing radiation and biological aging presents a landscape fraught with challenges, gaps, and shortfalls. Despite significant progress in understanding the effects of ionizing radiation and biological aging, several critical areas require further exploration and resolution.

**Dose–Response Relationships and Variability**: First and foremost is the challenge of establishing precise dose–response relationships, complicated by inherent variability in individual responses to ionizing radiation. This variability represents a significant gap in our understanding, as the molecular mechanisms underlying inter-individual differences in radiation response are not fully elucidated. Consequently, there is a shortfall in the development of personalized dosimetry models that consider genetic, epigenetic, and environmental factors, limiting accurate estimations of radiation-induced aging effects.

**Integration of Multi-omics Data**: As a recurring message on multi-omics, the integration of diverse datasets poses computational challenges, particularly in managing high-dimensional data and ensuring cross-platform compatibility. This challenge is exacerbated by a gap in our knowledge about the combinatorial effects of genetic, epigenetic, and transcriptomic changes following radiation exposure. Consequently, there is a shortfall in the development of robust computational tools that can comprehensively analyze integrated multi-omics data to reveal intricate molecular interactions.

**Temporal Dynamics of Multi-omic Modifications**: Another critical challenge is investigating the temporal dynamics of radiation-induced modifications. While these changes may evolve over time and interact with aging processes, there is a notable gap in the field regarding longitudinal studies tracking omic alterations post radiation exposure and their correlation with aging phenotypes. This knowledge shortfall impacts our understanding of whether certain changes persist, reverse, or accumulate over time, impeding the development of targeted interventions.

**Biomarkers for Radiation-Induced Aging**: Identifying reliable biomarkers for radiation-induced aging that can be translated into clinical or public health settings remains a formidable task. The gap in this context is represented by the limited validation and standardization of potential biomarkers, hindering their utility in assessing individual susceptibility to radiation-induced aging. This shortfall manifests in the absence of a consensus on a universal set of biomarkers that can effectively capture the complex and multifaceted aspects of biological aging following radiation exposure.

**Ethical Considerations and Radiation Exposure Limits**: Ethical considerations in studying radiation effects raise challenges in balancing the need for research with ethical standards, especially in longitudinal studies. The gap exists in inadequate guidelines on ethical considerations for such studies and potential long-term consequences for human subjects. This shortfall is compounded by a lack of established frameworks for translating research findings into policies that effectively regulate occupational, medical, and environmental exposure to ionizing radiation.

**Translation of Findings into Clinical Practice**: Translating research findings into actionable interventions or clinical strategies poses a substantial and typical challenge. The gap lies in the limited number of studies on the effectiveness of interventions targeting radiation-induced aging, hampering the development of therapeutic strategies. There is a shortfall in established protocols for incorporating radiation-induced aging assessments into clinical decision-making processes.

**Radiation in a Lifespan Context**: Considering the lifespan context of radiation exposure and its implications for aging furthers the challenge of conducting comprehensive longitudinal studies, which are logistically demanding. A gap exists in our understanding of how early-life radiation exposures impact aging trajectories later in life. This shortfall is underscored by incomplete knowledge about the cumulative effects of repeated radiation exposures over a lifetime on biological aging processes.

Addressing these challenges, filling existing gaps, and overcoming identified shortfalls in the study of the association between ionizing radiation and biological aging will undoubtedly require interdisciplinary collaboration, innovative methodologies, and sustained efforts within the field of biomedical engineering.

### 4.5. Future Developments

A recurring theme is that addressing one issue overlaps with many others. The main interest in ionizing radiation may be solved via a combination of other routes. An interest in another field may also offer a solution from the perspective of radiosensitivity. It comes down to the root cause. In this case, tackling indirect damage means dealing with oxidative stress. Whereas direct damage from radiation is extremely difficult to prevent because of penetrative power, a researcher could then look at repair mechanisms as a reactive approach to radiosensitivity.

One critical area of future development involves the advancement of radiation detection and monitoring technologies. Researchers are actively working on developing more sensitive and accurate dosimetry methods that can precisely measure radiation exposure, especially at low doses. Recent studies have explored novel approaches, such as using nanomaterials and miniaturized detectors, to enhance radiation monitoring capabilities. These advancements are essential for ensuring the safety of individuals exposed to radiation in various settings, from health care to space exploration. As the understanding of radiation-induced aging deepens, researchers are actively exploring radioprotective interventions that can mitigate the detrimental effects of radiation exposure. Recent studies have investigated the potential of various compounds, including antioxidants, radioprotective drugs, and natural extracts, to enhance the body’s resilience to ionizing radiation. These interventions aim to bolster DNA repair mechanisms, reduce oxidative stress, and minimize cellular damage caused by radiation. Additionally, advancements in personalized medicine may enable the development of tailored radioprotective strategies based on an individual’s genetic and epigenetic profiles.

With the growing interest in space exploration, particularly missions to Mars and beyond, space radiation research has gained prominence. Future developments in this field involve the assessment of radiation exposure risks during extended space missions and the development of effective shielding strategies. Recent studies have utilized computational models and experimental data from the International Space Station to evaluate the impact of space radiation on astronauts’ health, as well as secondary analysis of the lab-controlled environment to better characterize effects of space-level radiation levels [144,145]. These findings are crucial for designing spacecraft and protective measures to ensure the well-being of space travelers. The emerging field of precision medicine holds great potential for advancing rad–age association research. By integrating genomic, epigenomic, and multiomics data, researchers can develop personalized risk assessments for individuals exposed to radiation. Recent studies have explored the use of machine learning algorithms to predict an individual’s susceptibility to radiation-induced aging and age-related diseases. Precision medicine approaches aim to identify specific genetic variants and epigenetic modifications that modulate an individual’s response to radiation, enabling tailored interventions and therapies.

Implementing a multi-omics approach in future research exploring the association between ionizing radiation and biological aging demands a strategic integration of diverse high-throughput sequencing technologies and advanced computational methodologies. To effectively capture the temporal dynamics of molecular changes post radiation exposure, longitudinal studies employing next-generation sequencing (NGS) platforms are pivotal. NGS, known for its cost-effectiveness and high throughput, can simultaneously capture genomics, epigenomics, and transcriptomics data, unraveling the interplay between genetic variations, epigenetic modifications, and altered gene expression patterns in response to radiation. Moreover, the inclusion of single-cell sequencing techniques is essential to dissect the heterogeneity in cellular responses, providing a granular understanding of the dynamics at a single-cell resolution.

Epigenomic profiling will play a central role in unraveling radiation-induced molecular alterations. Techniques like bisulfite sequencing and chromatin immunoprecipitation sequencing (ChIP-Seq) are instrumental in assessing DNA methylation changes and mapping histone modifications, respectively. In parallel, transcriptomic analysis using differential gene expression and alternative splicing analysis methods uncovers genes with altered expression levels and reveals post-transcriptional regulatory changes post radiation. Complementing these, mass spectrometry-based proteomic analysis and metabolomic profiling offer insights into alterations in protein expression and metabolic pathways, providing a comprehensive picture of cellular responses to ionizing radiation.

For data integration and analysis, the application of advanced computational tools and systems biology approaches is imperative. Platforms like Galaxy and Bioconductor facilitate streamlined bioinformatics analyses, while software such as Cytoscape (https://cytoscape.org/) enables network-based visualization and exploration of intricate molecular interactions. Furthermore, machine learning algorithms, including feature selection and cross-validation techniques, may accelerate the identification of relevant omics features associated with radiation-induced aging and the building of predictive models.

In essence, the technical orchestration of a multi-omics approach in future research holds immense potential to unravel the complexities of ionizing radiation-induced biological aging. This integrative strategy, combining advanced sequencing technologies, computational methodologies, and ethical considerations, will not only deepen our understanding but also lay the groundwork for targeted biomedical engineering interventions in radiation-exposed populations.

Collaborative efforts and increased funding avenues are essential to drive future developments in rad–age association research. Recent initiatives, such as research proposals presented to governmental bodies, underscore the importance of addressing key research questions related to radiation exposure and aging. Collaborations between academia, government agencies, and industry partners can accelerate progress in this field and facilitate the translation of research findings into practical applications. A few research questions that have recently been presented to the US Senate include how cells detect and respond to low doses of radiation; responses at the molecular, cellular, tissue, and whole-body level; low-dose exposure response compared to high-dose response; and how updated techniques for data generation can be applied to new studies [146]. Programs tackling these questions are expected to integrate multi-omics research, create reproducible experiments with accessible datasets, or even partner with government organizations for the betterment of the general public. While research funding involving ionizing radiation (especially low-dose or -energy radiation) has increased in European countries and Japan, whereas funding in the United States has declined, totaling USD *$*210M from 2012 to 2016, with the DOE and NIH as primary contributors to related research. It is noted that even though radiation is a form of treatment in over 50% of cancer patients, the NIH allots 5% of its budget towards the topic [147].

## 5. Conclusions

This comprehensive review explored the profound effects of ionizing radiation on biological systems, shedding light on its role as a potent oxidative stressor with implications for estimating biological age. By dissecting the mechanisms of DNA damage and oxidative stress, we see potential parallels between radiation-induced damage and the natural aging process. This convergence highlights radiation as a powerful tool for investigating the relationship between radiation exposure and aging.

As we move forward, the pursuit of a precise recipe for measuring biological age continues to evolve, drawing from a diverse array of parameters and multi-omics approaches. The synergy between radiation research and aging studies holds the potential to revolutionize various fields, spanning from oncology to occupational health and space exploration. The comprehensive table of markers presented in this review is not merely a compilation; it represents a foundational resource that propels the scientific community toward a more nuanced and integrated understanding of the intricate interplay between radiation exposure and the aging process.

To propel our understanding further, the next crucial steps involve the execution of experiments with model organisms, capturing the multifaceted parameters that constitute biological age and facilitating the construction of predictive models. This iterative process, informed by the markers outlined in our comprehensive table, is poised to unlock new dimensions in our comprehension of the rad–age association.

## Figures and Tables

**Table 1:** Indicators of biological age.

Functional Biomarkers	
Cognitive [101,102]	Physical [103,104,105,106]
Memory	Grip strength
Decision reaction time	Walking speed and balance
Verbal fluency	Visual perception
	Height, weight/BMI
	Lung capacity
Organs and Tissues [107,108]	Cellular [109,110,111]
Brain size and sex	9 Hallmarks
Blood composition	of aging
Blood pressure	
Muscle mass	
Bone density	
**Psychological Well-Being Biomarkers**	
Hedonic [112]	Eudaimonic [113]
Happiness	Self-acceptance
Subjective well-being	Environmental mastery
Positive smotions	Positive relationships
	Personal growth
	Purpose in life
	Autonomy

## Data Availability

Not applicable.

## Abbreviations

The following abbreviations are used in this manuscript:

AI/ML	Artificial intelligence/machine learning
BA	Biological age
CA	Chronological age
DOE	Department of Energy
DSB	Double-strand break
MDPI	Multidisciplinary Digital Publishing Institute
NIH	National Institute of Health
PCA	Principal Component Analysis
ROS	Reactive Oxygen Species
SSB	Single-Strand Break
